# Has COVID-19 Modified the Weight of Known Systemic Inflammation Indexes and the New Ones (MCVL and IIC) in the Assessment as Predictive Factors of Complications and Mortality in Acute Pancreatitis?

**DOI:** 10.3390/diagnostics12123118

**Published:** 2022-12-10

**Authors:** Patricia Mihaela Radulescu, Dragos Virgil Davitoiu, Vlad Dumitru Baleanu, Vlad Padureanu, Dumitru Sandu Ramboiu, Marin Valeriu Surlin, Tudor Constantin Bratiloveanu, Eugen Florin Georgescu, Costin Teodor Streba, Razvan Mercut, Elena Irina Caluianu, Emil Tiberius Trasca, Dumitru Radulescu

**Affiliations:** 1 UMF Craiova Doctoral School, University of Pharmacy and Medicine Craiova, 200349 Craiova, Romania; 2 Department of General Surgery, Faculty of Medicine, ‘Carol Davila’ University of Medicine and Pharmacy, 020021 Bucharest, Romania; 3 Internal Medicine Department, Country Hospital of Craiova, University of Medicine and Pharmacy of Craiova, 200349 Craiova, Romania; 4 General Surgery Department, University of Medicine and Pharmacy of Craiova, 200349 Craiova, Romania; 5 Department of Pneumology, University of Pharmacy and Medicine Craiova, 200349 Craiova, Romania; 6 Department of Plastic and Reconstructive Surgery, University of Medicine and Pharmacy of Craiova, 200349 Craiova, Romania

**Keywords:** acute pancreatitis, IIC, cumulative inflammatory index, MCVL, average crepuscular volume−lymphocyte ratio, abscess, necrosis, pseudocyst, NLR, neutrophil-lymphocyte ratio

## Abstract

We aimed at evaluating the prognostic capacity of the inflammatory indices derived from routine complete blood cell counts in two groups of patients with acute pancreatitis from two different time periods, before and during the COVID-19 pandemic, when a high incidence of complications with surgical risk and mortality was found. Two new markers were introduced: the mean corpuscular volume to lymphocyte ratio (MCVL) and the cumulative inflammatory index (IIC), which were calculated at a baseline in the two groups of patients. Of the already established markers, none of them managed to effectively predict the complications with surgical risk and mortality, with a decrease of less than 50% in specificity in the peri-COVID group. The MCVL had the best prediction of complications with surgical risk in both the pre-COVID and peri-COVID groups, validated it as an independent factor by multivariate analysis. The IIC had the best prediction of mortality in both periods and was proven to be an independent factor by multivariate analysis. As the IIC predicted death best, we tested the occurrence of death and found that patients with PA who had an IIC > 12.12 presented a risk of death 4.08 times higher in the pre-COVID group and 3.33 times higher in the peri-COVID group. The new MCVL and IIC independent markers had a superior sensitivity and specificity in predicting surgical risk complications and, respectively, mortality in the group of patients with acute pancreatitis during the COVID-19 pandemic, which makes them widely applicable in populations with modified immune and inflammatory status. Conclusions: In patients with acute pancreatitis, MCVL has a significant predictive value regarding complications with surgical risk (abscess, necrosis, and pseudocyst), and the IIC has a significant predictive value for mortality.

## 1. Introduction

Globally, the incidence of acute pancreatitis is continuously increasing; in many countries, it is a significant health burden, affecting 34 people out of 100,000 per year [1]. The incidence of acute pancreatitis varies in Europe between 4.6 per 100,000 inhabitants in Albania and 100 per 100,000 inhabitants in Poland [2]. Depending on the biliary and alcoholic etiology of acute pancreatitis, the studies showed ratios of 6:1 in the Mediterranean countries of southern Europe [3], while the lowest ratio between gallstones and alcohol was less than 0.5:1 and was found mainly in Eastern European countries, including Romania [4]. The diagnosis of drug-induced acute pancreatitis is difficult to establish, with an incidence of less than 2%, and it is underreported due to the technical difficulty of diagnosis [5]. Most European studies carried out in the last five decades showed an increase in incidence by approximately 3–4% per year [6].

Acute pancreatitis is defined as an acute inflammatory condition that puts life at risk, ranging from a simple edema to necrosis, through the intraglandular activation of pancreatic enzymes. According to the Atlanta classification system, acute pancreatitis is divided into mild, moderate, and severe, depending on the severity [7]. In most cases, 80–90% have mild and moderate acute pancreatitis. On the other hand, 10–20% of cases have severe disease, where mortality is 30–50% [1]. Its evolution comprises two stages, the initial one in the first week, which is accompanied by organ failure, and the late one after the first week, which is accompanied by local complications, such as infected necrosis, abscess, or pseudocyst formation [8]. Improvements in early and accurate diagnosis, as well as the care of people with severe acute pancreatitis, led to a decrease in the overall mortality to 0.8%; still, this aspect, as well as the long-term effects, remains significant [9].

Coronavirus disease-19 (COVID-19) first occurred in Wuhan, China; it was declared a pandemic in March 2020 and subsequently became a major global health threat. Since 2021, over 100 million people from 210 countries have been confirmed to have been infected with the COVID-19 virus [10].

In Romania, the first case of infection with the new coronavirus was confirmed on 26 February 2020, and the first 3 deaths were recorded on 22 March 2020, reaching a maximum of 591 deaths per day on 2 November 2021. The virus is expected to remain active until 2024 even after adequate control measures, especially with the emergence of many mutants called variants of concern, such as B.1.1.7 (alpha), B.1.617.2 (delta), and B.1.1.529 (omicron) [11]. Liu et al. studied the damage to the pancreas by the SARS-CoV-2 infection and the association with ACE-2 receptors, which are slightly more expressed in the pancreas than in the lungs and may lead to the direct damage to the pancreas that was reported in 2% as non-severe, compared to 17% of the patients with severe COVID-19 [12].

To predict the prognosis and severity of PA, serum markers such as procalcitonin, C-reactive protein (CRP), Neutrophil CD64 Index, interleukin-6, and interleukin-8 were used, but they are expensive, barely available, and cannot accurately predict the prognosis and severity of PA [13,14].

For the past two decades, complete blood cell counts have routinely been performed, and they are relatively inexpensive and easy to perform. Neutrophils and monocytes are components of the innate immune system; lymphocytes are indicators of the adaptive immune response, and platelets, in addition to hemostasis, coagulation, and angiogenesis, are involved in the inflammatory reaction and innate immunity [15]. The hematological indices derived from the leukocyte count are: the neutrophil/lymphocyte ratio (NLR) based on the coexistence of leukocytosis and lymphopenia in the initial inflammatory response [16]; the derived neutrophil/lymphocyte ratio (dNLR); the monocyte/lymphocyte ratio (MLR), reflecting the body’s immune status, with a decrease indicating an immune dysfunction of the host; the platelet/lymphocyte ratio (PLR), which is an inflammatory marker of immune-mediated, metabolic, and prothrombotic diseases; the aggregate index of systemic inflammation (AISI), which is studied in lung diseases [17] and COVID-19 [18]; and the systemic inflammatory response index (SIRI) and the systemic inflammatory index (IIS), which are new inflammatory markers that are increasingly studied as biomarkers in several diseases, such as neoplastic [19], inflammatory, and infectious diseases such as acute pancreatitis [20] and COVID-19 [21].

Currently, there are few studies regarding their importance when taken separately in predicting severity and outcomes, and they were not analyzed together in predicting complications with surgical risk such as abscess, necrosis, pseudocyst, and mortality, either in the period before the pandemic or during the COVID-19 pandemic. Considering that, lately, there has been an emphasis on the identification of new biomarkers with improved predictive performances, we limited ourselves to the simple analysis of the peripheral blood upon admission to the hospital in the first 24 h, without performing any additional biological investigations. Following the observation of increased values of RDW and MCV upon hospitalization in the patients who died during hospitalization, we developed two new markers: the MCVL (the ratio between the average corpuscular volume and lymphocytes) and the IIC (cumulative inflammatory index). They reflect the numerical changes that occur at the level of red and white blood cells following an inflammatory process.

The aim of the study is to assess the values of NLR, PLR, MLR, dNLR, AISI, SIRI, SII, and the newly introduced MCVL and IIC as independent predictive factors in surgical risk complications and mortality in patients with AP and to test their accuracy in two time periods, before COVID-19 (pre-COVID) and during the COVID-19 pandemic (peri-COVID), when cell-mediated immunity is affected [22] and can decrease their predictability. The establishment of reliable predictive markers may help to prompt the initiation of medical management and thus obtain better outcomes for the patients with AP in terms of surgical risk complications and mortality. (Table 1)

## 2. Materials and Methods

### 2.1. Study Design

We retrospectively studied the consecutive PA cases from two academic medical centers, the Emergency County Clinical Hospital of Craiova and the Military Emergency Clinical “Dr. Stefan Odobleja” Hospital of Craiova, after obtaining the approval of the Ethics Commission of each unit. We used the hospital automation systems to collect patient data by ICD-10 diagnostic code (K85), including for the study cases that had enough data to be validated in their entirety.

**Figure 1:**
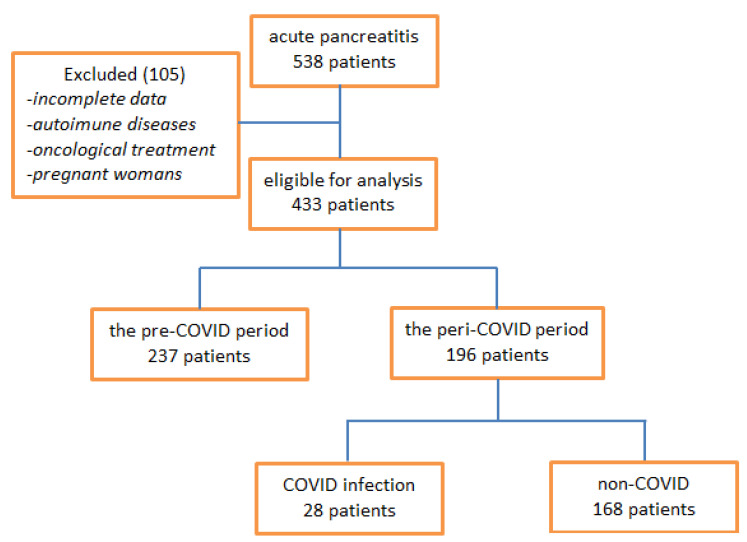
Flow chart of patient inclusion.

After applying the inclusion criteria, the data of 433 patients with acute pancreatitis admitted to the surgery service were analyzed; they were then divided into two groups, one before the pandemic, including patients admitted from 1 January 2018 to 26 February 2020 (pre-COVID), when the first case of COVID-19 was officially declared in Romania, and a second group that included patients from 27 February 2020 to 30 April 2022 (peri-COVID) (Figure 1). The data collected and considered were the following: demographic data (sex and age), the number of hours since the onset of symptoms, and the degree of severity obtained by correlating clinical and imaging data [23].

The result of the first blood test in the first 24 h after presentation was also taken into account and included: the number of white blood cells (WBC), the number of neutrophils (NEU), the number of lymphocytes (LYM), the number of monocytes (MON), the platelet count (PLT), the red cell distribution width (RDW), and the mean corpuscular volume (MCV). Having obtained the laboratory tests available after the first blood collection, we collected the data obtained from the blood cell count in the peripheral blood at admission, and we calculated the inflammation indices derived from the blood cell count as follows: the neutrophil–lymphocyte ratio (NLR) (neutrophils/lymphocytes); the derived neutrophil–lymphocyte ratio (dNLR) (neutrophils/(WBC-neutrophils)); the monocyte–lymphocyte ratio (MLR) (monocytes/lymphocytes); the platelet–lymphocyte ratio (PLR) (platelets/lymphocytes); the systemic inflammatory index (SII) (neutrophils ×platelets)/lymphocytes), and the systemic inflammatory response index (SIRI): (neutrophils × monocytes)/lymphocytes), AISI ((neutrophils × monocytes × platelets)/lymphocytes), MCVL (mean corpuscular volume/lymphocytes), and IIC ((mean crepuscular volume × width of erythrocyte distribution × neutrophils)/(lymphocytes ×1000)).

The etiological factors were obtained by examining the data recorded from the anamnesis (alcohol intake), the family history (autoimmune diseases), and the routine imaging examinations. Patients without an etiological factor were registered as “unknown”. Data such as number of hospitalization days, complications with surgical risk, and mortality were recorded. We included patients who had undergone procedures such as surgery for complications and medical therapy for AP treatment. The diagnosis of COVID-19 was established by real-time reverse transcription polymerase chain reaction (RT-PCR) nasopharyngeal swab testing. Patients who had a negative result in two tests were considered negative according to the recommendation of the Romanian Ministry of Health. Depending on the results, the patients in the peri-COVID group were divided into two groups, the COVID-19 (n = 28) and the non-COVID-19 (n = 168) group, with data comparison between these two groups.

### 2.2. Inclusion Criteria

The patients included in the study were those with the diagnosis of acute pancreatitis, confirmed biologically and by imaging according to the revised Atlanta Criteria [24], from 1 January 2018 to 30 May 2022, including the first blood analysis performed before starting medical treatment and those who had completed chemotherapy, radiotherapy, or combined treatment for various neoplastic conditions.

### 2.3. Exclusion Criteria

Patients excluded from the study were those with incomplete data at admission and a chronic treatment of oral corticosteroids and immunosuppressant drugs in the previous three months; those with incomplete oncological treatment; those with autoimmune diseases; and pregnant women.

### 2.4. Statistical Analysis

Data analysis was performed using SPSS version 25.0 (released by IBM Corp in 2017), with the establishment of descriptive statistics of the studied population.

The data were first tested for normality and homogeneity of variation.

The values that were normally distributed were expressed as ±SD, and categorical values were expressed as percentages. The independent t-test was performed to determine the effect of the inflammatory indices on complications or mortality.

The optimal value was established following the analysis of the characteristic ROC curve, determining a maximum normal limit in order to maximize the specificity and sensitivity of each index. Limits were set by maximizing the sum of the sensitivity and the specificity.

The results were expressed as mean value ± standard deviation for continuous quantitative variables. The quantitative variables were expressed in the form of proportions. The univariate statistical analysis was performed using hypothesis confirmation tests: the chi-squared test for qualitative variables and the Student t test for comparing quantitative variables with the homogeneity of variations in the Levene test, whose results are less affected by unequal group sizes [25]. To adjust for potential confounding effects, multivariate logistic regression analyses were performed, with 95% confidence intervals (CIs). We considered the results to be statistically significant if the *p* values of the two groups were <0.05, with a confidence interval of 95%.

## 3. Results

We retrospectively analyzed a total of 538 patients presented to the emergency room and diagnosed with acute pancreatitis between 1 January 2018 and 30 May 2022. Among these patients, we included in the study a total of 433 patients who met the inclusion criteria and on whom there were enough data from the laboratory analyses performed in the first 24 h after admission to the hospital.

The patients were divided into two groups; the first group was called pre-COVID and included patients admitted between 1 January 2018 and 3 March 2020 (n = 237), and the other group we called peri-COVID; it included patients admitted during the COVID-19 pandemic between 3 March 2020 and 5 May 2022 (n = 196).

The patients in the peri-COVID group were also divided into two groups: positive for COVID-19 (COVID-19) and negative for COVID-19 (non-COVID-19).

### 3.1. Characteristics of the Patients

Among the patients in the pre-COVID group, 56.5% were men and had a mean age of 57.57 ± 13.43 (n = 134), while the mean age of the female patients was 58.78 ± 19.89 years (n = 103) (Table 2). Regarding the etiology of pancreatitis, the most common was gallstones 82.2% (n = 195), followed by alcohol intake 12.6% (n = 30), and unknown etiology 4.64% (n = 11). According to the Atlanta classification, 67.5% (n = 81) had a mild form, 46.7% (n = 86) had a moderate form, and 54.3% (n = 70) had a severe form of PA. Regarding the complications with surgical risk, the was total was 11.8% (n = 28), represented by abscess 3.8% (n = 9), necrosis 3.8% (n = 9), and pancreatic pseudocyst 4.2% (n = 10); in 6.8% (n = 16) of the cases, there were complications (6.8%) requiring surgical intervention. The mean duration of hospitalization was 14 ± 16.55 days, and discharge occurred in 10.1% of the patients (n = 24).

In the peri-COVID group, 46.4% were men and had a mean age of 62.19 ± 17.21 (n = 91), while the mean age of the female patients was 65.55 ± 15.45 years old (n = 105). Regarding the etiology of pancreatitis, the most common was gallstones 82.6% (n = 162), followed by alcohol intake 11.2% (n = 22) and unknown etiology 6.1% (n = 12). According to the Atlanta classification, 32.5% (n = 39) had a mild form, 53.3% (n = 98) had a moderate form, and 45.7% (n = 59) had a severe form. Regarding the complications with a surgical risk, there were 40 in total, represented by abscess 3.8% (n = 15), necrosis 3.8% (n = 20), and pancreatic pseudocyst 2.6% (n = 5), and 9 cases with complications (4.6%) requiring surgical intervention. The mean duration of hospitalization was 11.37 ± 7.58 days, and discharge occurred in 18.4% of the patients (n = 36) (Table 2).

Sixty-seven point nine percent of the PA patients with a confirmed COVID-19 infection were men and had a mean age of 47 ± 15.61 (n = 19), while the mean age of the female patients was 48.44 ± 20.71 years old (n = 9) (Table 2). Regarding the etiology of pancreatitis, the most common was gallstones 46.4% (n = 13), followed by alcohol intake 28.6% (n = 8) and unknown etiology 25% (n = 7). According to the Atlanta classification, 10.7% (n = 3) had a mild form, 46.4% (n = 13) had a moderate form, and 42.9% (n = 12) had a severe form. Regarding the complications with surgical risk, the total was 36% (n = 10), represented by abscess 10.7% (n = 3), necrosis 17.9% (n = 5), and pancreatic pseudocyst 7.1% (n = 2), and there was only one case with complications (3.6%) requiring surgical intervention. The mean duration of hospitalization was 12.36 ± 8.89 days, and discharge occurred in 42.9% of patients (n = 12).

Comparing the data from the pre-COVID and peri-COVID groups, the mean age had a statistically significant higher value in the peri-COVID group, and the male to female ratio was higher in the pre-COVID group (1.3:1) and was also statistically significant. We found no statistically significant difference between the ratios of etiology in the two groups. According to the revised Atlanta classification, a slight increase in the moderate pancreatitis cases and a slight decrease in the mild cases in the peri-COVID group without statistical significance were observed. The mean length of hospitalization was statistically significantly shorter in the peri-COVID group; however, the number of complications and the mortality were statistically significantly higher in the peri-COVID group (Table 2).

Comparing the data of the AP patients with the COVID-19 infection (the COVID-19 group) with those with non-COVID-19 AP in the peri-COVID group, the mean age was statistically significantly lower in the COVID-19 group, the male–female ratio being high (2.1:1), with high significant statistics. The ratio of alcoholic and unknown etiology was statistically significantly increased compared to the non-COVID-19 patients, for whom biliary etiology also fell below half. The average duration of hospitalization was similar in the two groups but with a statistically significant increase in the rate of complications with surgical risk, which almost doubled in the COVID-19 group, with a halving of the operability. Mortality was almost three times higher in the group of patients with AP and COVID-19 compared to the non-COVID-19 group (Table 3).

### 3.2. Biological Parameters at Admission

Comparing the biological parameters taken separately from the pre-COVID and peri-COVID groups, only one statistically significant difference was found among the MCVs which was higher among peri-COVID patients. In the case of the patients in the COVID-19 group compared to the non-COVID-19 patients, the value of the neutrophils was significantly higher in the COVID-19 group (13.86 ± 8.94 vs. 10.57 ± 5.60, *p* = 0.040). Moreover, the MCV and RDW were significantly higher in the COVID-19 group (103.46 ± 4.7 vs. 91.35 ± 6.17, *p* < 0.001) and (14.57 ± 1.9 vs. 13.27 ± 1.26, *p* < 0.001).

Regarding the variation of the biological parameters in the complications of the patients in the pre-COVID group, the values of the group of patients with complications and the group of patients without complications were compared, and statistically significant variations were recorded only among the lymphocytes (1.16 ± 0.39 vs. 1.72 ± 0.79, *p* < 0.001).

In the peri-COVID group, following the comparison of the values from the group of patients with complications and the group of patients without complications, statistically significant variations were recorded only among the MCVs (99.63 ± 5.12 vs. 92.02 ± 7.31, *p* < 0.001).

Regarding the variation of the biological parameters in the mortality of the patients in the pre-COVID group, the values of the group of patients who died were compared with the group of patients who survived, and statistically significant variations were recorded among the neutrophils (14.38 ± 7.48 vs. 10.41 ± 5.53, *p* = 0.002), lymphocytes (0.91 ± 0.41 vs. 1.74 ± 0.94, *p* < 0.001), and RDW (14.5 ± 1.3 vs. 13.09 ± 1.88, *p* < 0.001) (Table 3).

### 3.3. Levels of Inflammatory Indices Calculated at Admission

Although the total leukocyte counts were not similar in the pre-COVID and peri-COVID groups with or without COVID-19 infection, there were significant variations in the inflammatory indices.

Comparing the inflammatory indices in the pre-COVID and peri-COVID groups, a statistically significant higher value was found among the NLR (8.46 ± 6.01 vs. 10.24 ± 8.61, *p* = 0.015), MLR (0.61 ± 0.38 vs. 0.78 ± 0.77, *p* = 0.005), dNLR (4.6 ± 2.93 vs. 6.08 ± 6.68, *p* = 0.006), MCVL (70.26 ± 37.17 vs. 90.65 ± 67.15, *p* < 0.001), and IIC (10.73 ± 8.71 vs. 13.03 ± 10.86, *p* = 0.006).

Comparing the index values from the COVID-19 group and the non-COVID-19 group, a statistically significantly lower value was found among the NLR (7.85 ± 6.17 vs. 10.64 ± 8.91, *p* = 0.045), MCVL (11.04 ± 8.99 vs. 13.36 ± 11.31, *p* =0.029), and IIC (11.04 ± 8.99 vs. 13.36 ± 11.31, *p* < 0.001) (Table 4).

The differences in index values between the patients with complications and those without complications in the pre-COVID group showed significant increases in the values among the PLR (206.73 ± 108.62 vs. 154.13 ± 111.95, *p* < 0.001), AISI (3676.43 ± 4063.20 vs. 1661.20 ± 2747.85, *p* = 0.016), SIRI (11.73 ± 8.02 vs. 7.23 ± 8.01, *p* = 0.006), and MCVL (13.01 ± 6.75 vs. 13.01 ± 6.75, *p* = 0.028) (Table 5).

The differences in index values between patients with complications and those without complications in the peri-COVID group showed significant increases in values only among the IICs (17.20 ± 10.28 vs. 12.20 ± 10.73, *p* = 0.009).

The differences in the values of the indexes between the deceased patients and those alive in the pre-COVID group showed significant increases in the values among NLR (17.60 ± 7.15 vs.7.43 ± 4.9, *p* < 0.001), PLR (227.36 ± 119 vs. 152.8 ± 109.63, *p* < 0.002), MLR (9.96 ± 0.45 vs. 0.57 ± 0.35, *p* < 0.001), dNLR (8.06 ± 4.82 vs. 4.28 ± 2.36, *p* = 0.001), AISI (3891.86 ± 4342.95 vs. 1674.77 ± 2727.18, *p* = 0.022), SIRI (14.32 ± 9.69 vs. 7.02 ± 7.61, *p* = 0.001), SII (3542.55 ± 2775.46 vs.1682.57 ± 1807.43, *p* = 0.004), MCVL (117.88 ± 52.93 vs. 64.89 ± 30.8, *p* < 0.001), and IIC (22.62 ± 10.55 vs. 8.99 ± 7.31, *p* < 0.001) (Table 5).

Among the peri-COVID patients, the differences in the index values between the deceased and living patients showed significant increases in NLR (14.86 ± 7.72 vs. 9.20 ± 8.49, *p* < 0.001), MLR (1.07 ± 0.72 vs. 0.71 ± 0.77, *p* = 0.011), SIRI (12.98 ± 9.39 vs. 8.45 ± 11.16, *p* = 0.025), MCVL (146.64 ± 104.99 vs. 78.05 ± 47.27, *p* < 0.001), and IIC (12.12 ± 9.24 vs. 10.99 ± 9.86, *p* < 0.001) (Table 6).

### 3.4. The Predictive Values of Inflammatory Indices in Terms of Complications with Surgical Risk

Receiver operating characteristic (ROC) curve analysis was used to determine the predictive points of the marker levels for complications and mortality in the pre-COVID and peri-COVID groups.

**Figure 2:**
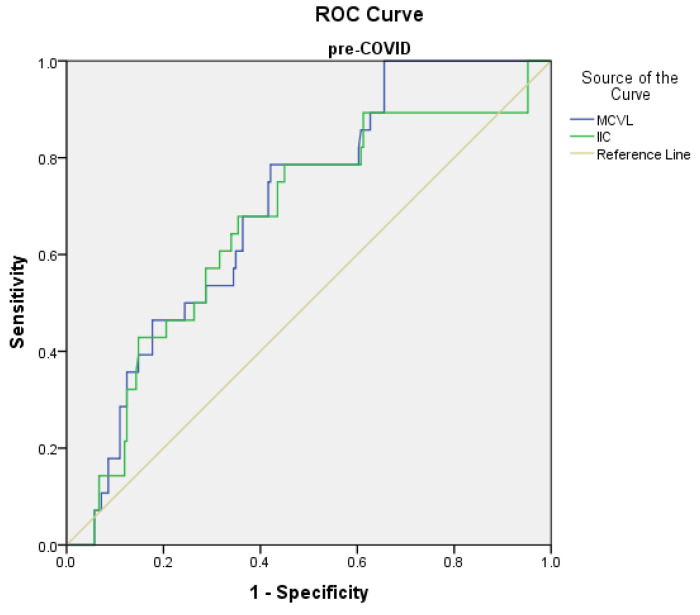
Receiver operating characteristic (ROC) curve for logistic regression models of inflammatory markers in relation to complications in the pre-COVID group.

The prediction of complications in the pre-COVID group using the ROC curve showed the significant results sustained by a specificity of over 50% on MLR, MCVL, and IIC, with the following values: the cutoff value of the MLR level was found to be >0.66 with 78.6% sensitivity and 69.9% specificity (AUC = 0.719, *p* < 0.001); MCVL was found to be >64.89 with 78.6% sensitivity and 56.8% specificity (AUC = 0.6 97, *p* = 0.001); IIC was found to be >8.41 with 78.6% sensitivity and 55.8% specificity (AUC = 0.663, *p* = 0.005) (Figure 2).

**Figure 3:**
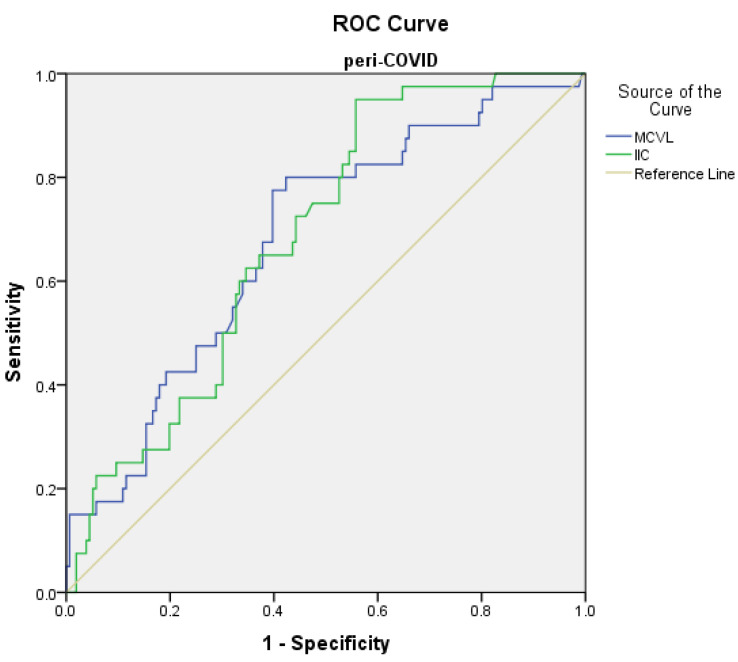
Receiver operating characteristic (ROC) curve for logistic regression models of inflammatory markers related to complications in the peri-COVID group.

The prediction of complications in the peri-COVID group using the ROC curve showed the following significant values (Figure 3): MCVL was found to be >78 with 80% sensitivity and 60.3% specificity (AUC = 0.681, *p* < 0.001), and IIC was found to be >10.51 with 72.5% sensitivity and 52.6% specificity (AUC = 0.686, *p* < 0.001) (Table 7).

### 3.5. The Predictive Values of Inflammatory Indices in Mortality

**Figure 4:**
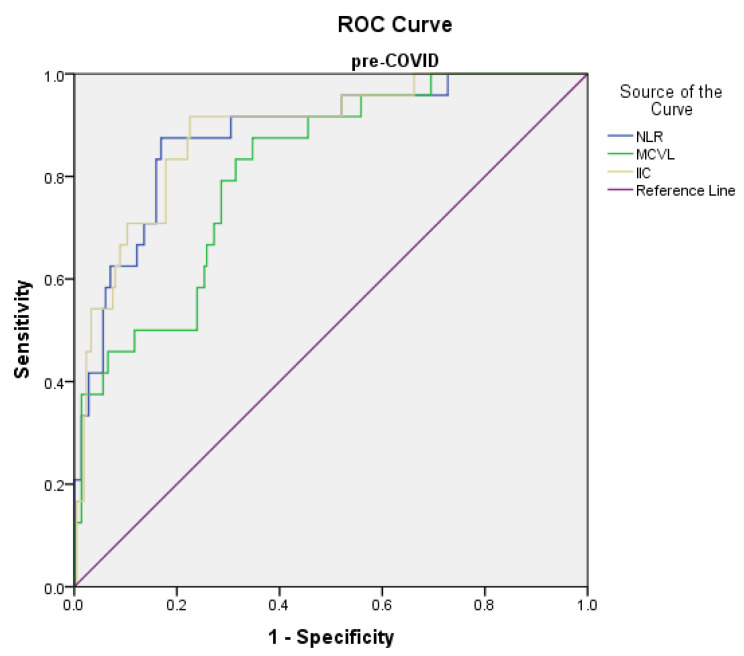
Receiver operating characteristic (ROC) curve for logistic regression models of inflammatory markers related to death in the pre-COVID group.

**Figure 5:**
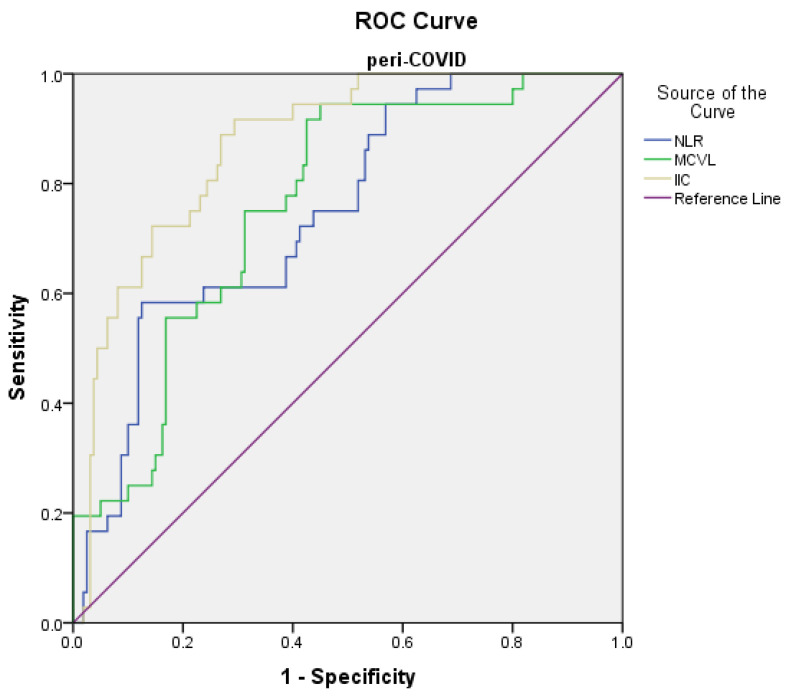
Receiver operating characteristic (ROC) curve for logistic regression models of inflammatory markers related to death in the peri-COVID group.

The prediction of the risk of death in the pre-COVID group using the ROC curve showed the following significant results: the cutoff value of the NLR level was found to be >11.01 with 91.7% sensitivity and 80.5% specificity (AUC = 0.833, *p* < 0.001); the cutoff value of the MCVL level was found to be >72.14 with 91.7% sensitivity and 67.6% specificity (AUC = 0.817, *p* < 0.001); and the cutoff value of the IIC level was found to be >13.29 with 91.7% sensitivity and 78.6% specificity (AUC = 0.887, *p* < 0.001) (Figure 4). The prediction of the risk of death in the peri-COVID group using the ROC curve showed the following (Figure 5): the cutoff value of the NLR level was found to be >5.93 with 91.7% sensitivity and 42.5% specificity (AUC = 0.743, *p* < 0.001); PLR, MLR, dNLR, AISI, SIRI, SII had the area < 0.700; the cutoff value of the MCVL level was found to be >74.9 with 94.4% sensitivity and 57.5% specificity (AUC = 0.762, *p* < 0.001); and the cutoff value of the IIC level was found to be >12.12 with 91.7% sensitivity and 72.5% specificity (AUC = 0.870, *p* < 0.001) (Table 8).

### 3.6. Univariate and Multivariate Analysis of Predictive Factors for Complications

The univariate analysis of the inflammatory markers was performed using the optimal cutoff obtained after the ROC curve analysis.

The univariate analysis results indicated that in the pre-COVID group there was a significant association of the complications with the lymphocytes: PLR > 97.3, MLR > 0.66, SIRI > 3.56, MCVL > 64.89, and IIC > 8.41. In the multivariate analysis, the value of the lymphocytes (*p* = 0.032) and of the inflammatory markers MCVL > 64.89 (OR 3.52, 95% CI 1.52–8.13, *p* = 0.003), and IIC > 8.41 (OR 5.56, 95% CI 2.87–10.95, *p* = 0.049) maintained a significant association with the complications (Table 9).

In the peri-COVID group, in the univariate analysis, the complications were associated with the area, proteins, ALT, MCV, MCVL > 78, and IIC > 10.51. The multivariate analysis showed a significant association of the complications with the area (*p* = 0.027) and ALT (*p* = 0.026) and among the inflammatory markers only with MCVL > 10.51 (OR 4.22, 95% CI 1.46–12.14, *p* = 0.008) (Table 10).

### 3.7. Univariate and Multivariate Analysis of Predictive Factors for Mortality

The univariate analysis indicated that in the pre-COVID group mortality was associated with levels of urea, BT, neutrophils, lymphocytes, NLR > 11.01, PLR > 102.88, dNLR > 3, SIRI > 4.26, SII > 906.21, MCVL > 72.14, and IIC > 13.29. The multivariate analysis showed a significant association of mortality with the levels of urea (*p* = 0.013), BT (*p* = 0.009), neutrophils (*p* = 0.025), and lymphocytes (*p* = 0.013) and among the inflammatory markers with NLR > 11.01 (OR 20.10, 95% CI 3.12–129.42, *p* = 0.002) and IIC > 13.29 (OR 18.71, 95% CI 2.60–134.52, *p* = 0.004) (Table 11).

In the peri-COVID group, in the univariate analysis, mortality was associated with protein levels, urea, creatinine, BT, INR, COVID-19, Hb, Ht, neutrophils, lymphocytes, RDW, NLR > 5.39, MCVL > 79.4, and IIC > 12.12. The multivariate analysis showed a significant association of mortality with the level of protein (*p* = 0.009), urea (*p* = 0.015), creatinine (*p* < 0.001), BT (0.003), infection with COVID-19 (0.001) and neutrophils (0.002) and among the inflammatory markers with NLR > 5.39 (OR 10.24, 95% CI 1.29–81.17, *p* = 0.028), MCVL > 79.4 (OR 8.92, 95% CI 5.21–141.58, *p* = 0.041), and IIC > 12.12 (OR 27.94, 95% CI 3.57–218.58, *p* = 0.002) (Table 12).

### 3.8. Results of Pearson Chi-Square Test

According to the results obtained in the multivariate analysis, we performed the Pearson chi-square test to evaluate the correlation between the MCVL and the complications with surgical risk and the IIC with mortality, which had a value of *p* < 0.001 in both cases. We also calculated for the MCVL the risk of developing complications, which is 1.85 times in the pre-COVID group and 0.37 times in the peri-COVID group at the cutoff values of 64.89 and 78, respectively. For IIC, the risk of death was 4.33 times in the pre-Covid group and 3.33 times in the peri-Covid group at the cutoff values of 13.29 and 12.12, respectively (Table 13).

## 4. Discussions

The present study investigates the predictability of surgical risk complications and mortality in patients with AP and the influence of the SARS-CoV-2 infection on it. For this, we used inflammation markers that were studied separately in the literature, without comparative studies before and during the COVID-19 pandemic. Moreover, in addition to the existing inflammatory markers, we introduced two new inflammation markers that better reflect the inflammation-induced changes to white and red cells in the peripheral blood. Wang et al. were the first to describe nine cases of acute pancreatitis in a series of 52 patients with COVID-19 [26].

The pandemic led to global chaos, particularly in the healthcare system where it caused sudden interruptions in the provision of healthcare to all patients across the country, caused by the relocation of workforces from healthcare facilities to treating patients with COVID-19 [27] or the panic instilled in the population that led to a decrease in trust in the medical act with a therapeutic [28] or in a preventive purpose such as vaccination [29]. The number of non-COVID-19 acute emergency cases decreased, a fact reflected by the low number of emergency presentations and the number of abdominal CT scans performed [30]. Moreover, a negative impact of COVID-19 was found in patients who had risk factors for diseases or already suffered from life-threatening diseases such as AP, with some hesitation from the patient to initially see a doctor because of the associated risk of exposure to the virus [31], a condition also found in our study, with 196 patients with AP vs. 237 who presented themselves in the pre-COVID period. There was also a decrease in the number of patients who presented with a mild form, from 34% to 19.8% according to the Atlanta classification, a fact explained by the fact that sometimes the patients resorted to self-medication by using the personal medicines of another family member prescribed by a doctor for chronic or recurrent diseases or symptoms [27]. There was a slight increase in the mild and severe forms and an increase in the time elapsed between the onset of symptoms and hospital presentation, resulting in an increase in the disease severity, ultimately causing the patient to present himself in the hospitalization unit with a more severe form of the disease.

The infection with COVID-19 affects the lungs as the main organ of the infection, but in cases with mild, moderate, and severe infection, gastrointestinal signs and symptoms were also reported, which included nausea, diarrhea, anorexia, abdominal pain, belching and vomiting [32], which led to some cases already hospitalized with COVID-19 with digestive symptoms refractory to treatment to be diagnosed with AP, which was classified as being of unknown etiology. In the peri-COVID group, a slight increase in the number of cases of PA of unknown etiology was observed, and more than half of them were patients in the COVID-19 group.

Acute pancreatitis is a common disease that has a sudden onset, rapid progression, and high mortality and morbidity; therefore, as soon as patients are diagnosed with this disease, their condition should be monitored as quickly as possible and with maximum accuracy. The need to anticipate their prognosis must lead to a treatment of the real suffering, which is sometimes masked by the associated conditions. As inflammatory mediators play a significant role in the occurrence of PA, various inflammatory markers were recently used to assess the predictive value of the severity and mortality of patients with PA.

In our study, we investigated the value of the known markers NLR, PLR, MLR, dNLR, AISI, SIRI, and SII and the newly introduced MCVL and IIC as independent predictive factors of surgical risk complications and mortality in patients with AP; we tested their accuracy in two time periods, namely before COVID-19 and during COVID-19. Their cutoff points for complications with surgical risk and mortality were observed using the ROC curve.

We did not use other serum predictive markers such as C-reactive protein, procalcitonin, interleukin-6, and interleukin-8 because there are numerous studies in which they cannot accurately predict the prognosis and severity of PA [13], and they were not used in our case on a wide scale.

Complications with surgical risk that can put life at risk are abscess, necrosis, and pancreatic pseudocyst. The pancreatic abscess is a complication that occurs particularly in patients with acute pancreatitis and is defined as a circumscribed intraabdominal collection of pus that is usually located near the pancreas and is caused by bacterial colonic translocation [33]. In AP patients whose abdominal symptoms do not improve after drug treatment and who begin to develop systemic inflammatory response syndrome, intraabdominal infection should be suspected and may require surgical treatment depending on the patient’s condition. In our case, a doubling of the incidence of abscesses, from 3.8% to 7.7%, was observed in the peri-COVID period with a higher incidence among COVID-19 patients, which was probably explained by their increased susceptibility to secondary infections, a fact reported in several reviews and meta-analyses [34,35] (Table 3).

Pancreatic necrosis is one of the serious complications of AP, with a significant proportion of these patients requiring surgical intervention. The main cause of death in a patient with pancreatic necrosis is infection of the necrotic tissue, which is a poor prognostic factor [36]; thus, pancreatic necrosis without infection has a mortality rate of approximately 15%, while patients with infected necrosis have a mortality rate of between 30 and 39% [37]. In our study, an increase in the number of cases of pancreatic necrosis was observed from 3.8% to 10.2%, a fact explained by the doubling of the incidence among patients with COVID-19 and PA, from 8.9% to 17.9%, possibly caused by the severity of these cases.

A pancreatic pseudocyst is a localized fluid collection that is rich in amylase and other pancreatic enzymes and is surrounded by a wall of fibrous tissue that is not lined by epithelium [38]. The treatment of a pancreatic pseudocyst can consist of open surgical drainage, endoscopic drainage, and percutaneous drainage. Surgical treatment is still used as a treatment option and involves internal drainage in the stomach, duodenum, and jejunum [39]. Endoscopic drainage did not show any significant difference between the success rates of surgical and endoscopic treatment, adverse events, and pseudocyst recurrence [40]. Percutaneous therapy showed a higher failure rate, a higher mortality and morbidity rate, and patients required more days of long-term hospitalization compared to those treated with surgery [41]. In our study, the incidence of the pancreatic pseudocyst was higher in the pre-COVID group.

Although, overall, the rate of complications with surgical risk was higher in the peri-COVID group, 68.7% occurred in patients in the COVID-19 group, with a rate of surgery reduced by half due to the contraindication of performing surgery in the context of already established multiorgan failure. Cases of severe AP and COVID-19 are characterized by a cytokine storm that ultimately leads to multiorgan failure and increased mortality.

Liu et al. studied the damage to the pancreas by the SARS-CoV-2 infection and the association with ACE-2 receptors, which are slightly more expressed in the pancreas than in the lungs, possibly leading to the direct damage to the pancreas that was reported in 2% of non-severe patients, compared to 17% of patients with severe COVID-19 [12].

The global mortality of AP is approximately 10–15%, reaching 30–50% in severe AP [1,42]. In our study, we recorded a mortality of 10.1% in the pre-COVID group, with an increase to 18.4% in the peri-COVID group, where a mortality of 42.9% was recorded among patients with a COVID-19 infection and AP.

The neutrophil–lymphocyte ratio (NLR) reflects the systemic inflammatory state and has a prognostic value in cardiovascular diseases, autoimmune diseases, tumors, and other infectious diseases [43,44,45]. Although, until now, the exact limit for NLR was missing, Forget et al. established that the normal values of NLR in a healthy, non-geriatric adult population is between 0.78 and 3.53 [46]. The first study that analyzed the relationship between NLR and the outcomes in patients with acute pancreatitis was conducted by Yao J. [47] in 2011. In a study by Jeon and Park, it was shown that high basic NLR was associated with severe PA and organ failure [48]. Azab et al. found that NLR was increased in PA patients, and they also reported that NLR was superior to total WBC or individual neutrophil or lymphocyte counts in predicting ICU admission and mortality for PA [49]. In another study that assessed the severity of PA, the NLR cutoff was >4.7, with a sensitivity of 90% and a specificity of 22% [13] In this study, regarding the constitution of the complications with surgical risk in the pre-COVID vs. peri-COVID period, the NLR had a cutoff of >5.58 vs. 6.41, with a sensitivity and specificity of 78.6% and 42.2% vs. 80% and 44.9%. The prediction of the complications was significant, but the specificity in both groups remained below 50%.

Regarding mortality, in the pre-COVID vs. peri-COVID period the NLR had a cutoff >11.01 vs. 5.93, with a sensitivity and specificity of 91.7% and 80.5% vs. 91.7% and 42.5%. The prediction of mortality was statistically significant in both groups, but the specificity dropped dramatically in the peri-COVID group, below 50%, with a halving of the cutoff value, making it an inapplicable marker in groups with impaired immunity due to other superimposed infectious conditions with PA.

In recent years, studies have shown that platelets and lymphocytes play a critical role in the inflammatory process; therefore, in recent research, the platelet lymphocyte ratio (PLR), which is a new inflammatory factor, received research attention, proving that it can be a good indicator of inflammation in multiple acute cardiac and renal diseases [50] and in acute pancreatitis [36]. Thrombocytopenia is related to chronic liver disease due to the impaired production of platelets and the decreased hepatic synthesis of thrombopoietin [51]; therefore, PLR can vary depending on liver function, but also on systemic inflammation. Increased NLR and PLR values have been associated with inflammatory conditions, and poor results in severe PA are explained by uncontrolled SIRS and its progression to multiple organ dysfunction syndrome [13]. Regarding the constitution of the complications with surgical risk in the pre-COVID vs. peri-COVID period, PLR had a cutoff of >97.3 vs. 140.2, with a sensitivity and specificity of 89.3% and 34.5% vs. 72.5% and 50.6%. The prediction of the complications was significant, but the specificity was low in the pre-COVID group, and the differences between the cutoff values were large. In terms of mortality, in the pre-COVID vs peri-COVID period PLR had a similar cutoff but with a specificity below 40%.

The monocyte to lymphocyte ratio (MLR) is the absolute number of monocytes divided by the absolute number of lymphocytes and is a hematological and inflammatory parameter. The MLR is associated with various infectious and inflammatory conditions as well as with the systemic inflammatory response, which reflects the immune status of the diseases [52,53]. The monocyte–lymphocyte ratio (MLR) was associated with adverse outcomes in colorectal and urological cancers [54,55]. Regarding the formation of complications with surgical risk in the pre-COVID vs. peri-COVID period, the MLR had a cutoff of >0.66 vs. 0.53, with a sensitivity and specificity of 78.6% and 69.9% vs. 75% and 49.4%. The prediction of complications was significant, but the specificity was slightly decreased in the peri-COVID group, without large differences between the cutoff values, which makes it a faithful marker in the prediction of complications. Regarding mortality, in the pre-COVID vs. peri-COVID period the MLR had a similar cutoff, but with a specificity below 40%.

The derived neutrophil lymphocyte ratio (dNLR) was studied in the evaluation of the prognosis of patients with metastatic disease regardless of the treatment followed [56], proving that it has a prognostic value similar to that of the classic NLR. It reflects the increase in the number of neutrophils and a relative decrease in the number of lymphocytes, suggesting that inflammatory reactions are dependent on neutrophils. Regarding the formation of complications with surgical risk in the pre-COVID vs peri-COVID period, the dNLR predicted the formation of complications and mortality, but with a specificity below 50%.

AISI, SIRI, and SII are considered to reflect the immune and inflammatory status, and a link between them and the risk of mortality in different types of cancer [57], autoimmune diseases [58], and infectious diseases [21] has been observed.

The aggregate index of systemic inflammation (AISI) had statistical significance in the prediction of complications only in the pre-COVID group, but with a very low specificity of less than 10%. The AISI also predicted mortality only in the pre-COVID group, but with a specificity below 50%.

The systemic inflammatory response index (SIRI) had statistical significance only in both groups with regard to the prediction of complications and mortality, but with a sensitivity below 50%.

The systemic immuno-inflammatory index (SII), derived from neutrophils, lymphocytes, and platelets, was used for the first time in 2014 in evaluating the prognosis of hepatocellular carcinoma [59]. A recent study showed that the SII was more sensitive than the NLR and PLR in distinguishing between mild and severe PA [20]. The SII was statistically significant in predicting complications and death only in the pre-COVID group, but with a sensitivity below 50%.

Red cells (RBC), which are produced in the bone marrow, are the most common type of cells in the blood [60] and have the function of supplying oxygen to organs and tissues. The MCV is the average volume of an RBC and is not only an indicator of anemia but is also a marker of chronic inflammation [61], being found to be associated with heart failure, diabetes, vascular accident, and venous thromboembolism [62]. Moreover, an increased MCV is an indicator of the deterioration of liver function, with macrocytosis being found in various liver diseases that lead to changes in the lipid composition of the RBC membrane [63].

The impact of a massive increase in the relative mass of RBC is an increase in the viscosity of whole blood, primarily by increasing the number of particles per unit of blood volume and, therefore, increasing the peripheral resistance to blood flow [64]. Viscosity is defined as the ratio between the shearing effort and the shear rate and is a risk factor for deep venous thrombosis, creating a procoagulant state through thrombocytosis and increasing the concentration of the circulating tissue factor [65]. Mean corpuscular volume is considered to have qualitative effects on blood rheology, rather than quantitative effects, such as hematocrit [66].

MCV alone has no role as a biomarker of severity in patients with AP, although it was found to have a value greater than 100 in the patients who died in the peri-COVID group. Its correlation with lymphocytes, whose low value shows a diminished initial immune response of the host [67], led to the establishment of a new prognostic marker of the severity of patients with PA, which we called the MCVL ratio, which was obtained by dividing the absolute number of the erythrocyte MCV by the absolute number of lymphocytes. Regarding the occurrence of complications with surgical risk in the pre-COVID vs. the peri-COVID period, the MCVL had a cutoff of >64.89 vs. >78, with a sensitivity and specificity of 78.6% and 56.8% vs. 80% and 60.3%. The prediction of complications was significant, with the best values among all the studied markers. The MCVL also proved its superiority in predicting mortality in the pre-COVID vs. peri-COVID period with a cutoff of >72.14 vs. 74.9, with a sensitivity and specificity of 91.7% and 67.6% vs. 91.7% and 57.3%; these are close values, which makes it useful in both types of groups.

Regarding the prediction of complications in the peri-COVID group, the multivariate analysis showed that the MCVL threshold value of 78 is an independent factor for the prediction of complications, with a slight decrease in the OR from 4.84 to 4.22.

The erythrocyte distribution width parameter (RDW) (the coefficient of variation of the RBC volume) is calculated after a routine complete blood count (CBC) and is the ratio between the standard deviation of the RBC volume and the mean corpuscular volume, which is useful in quantifying the heterogeneity of the RBC volume (anisocytosis). In addition to the association with a series of cardiovascular diseases, cancer, and diabetes [68], studies have reported an increased RDW in critically ill patients, making it a strong and independent risk factor for sepsis and septic shock mortality [69,70].

In our study, the RDW value was significantly higher in the group of patients who died, both in the pre-COVID and peri-COVID groups, which was confirmed by a cross-sectional study conducted in 2014 [47] by Yao J. et al. Finding these, we developed another marker to sum up the RBC changes that can occur in an acute or chronic inflammation, which is reflected by the MCV and RDW values, the numerical changes of the neutrophils that are elements of the innate immune system and of the lymphocytes that are indicators of the adaptive immune response.

Neutrophils have a key role in the destruction of local tissues and in the development of systemic complications of severe PA [71].

Lymphopenia may also be associated with the severity of PA, and some studies show that the impairment of cellular immunity caused by the apoptosis of peripheral lymphocytes may be related to the subsequent development of infectious complications in PA [72].

We called this marker the cumulative inflammatory index (IIC), and it was obtained by multiplying the MCV with the RDW and with the absolute number of neutrophils, all divided by the absolute number of lymphocytes multiplied by 1000 to obtain an index that is easy to use and remember.

In the complications with surgical risk in the pre-COVID vs. peri-COVID period, the IIC had a cutoff of >8.41 vs. 10.51, with a sensitivity and specificity of 78.6% and 55.8% vs. 72.5% and 52.6%. The prediction of complications was significant, with a specificity over 50% in both groups. The IIC proved to be the marker that best predicted mortality in the pre-COVID vs peri-COVID period with a cutoff of >13.29 vs. >12.12, with a sensitivity and specificity of 91.7% and 78.6% vs. 91.7% and 72.5%; these values are close to the cutoff values, making it useful in both groups.

Moreover, the IIC threshold value of 12.12 was significant in the prediction of mortality in the peri-COVID group, and in the multivariate analysis there was a slight decrease in the OR from 29 to 27.49, thus demonstrating that it is a strong independent factor for the prediction of mortality in the peri-COVID group.

**Figure 6:**
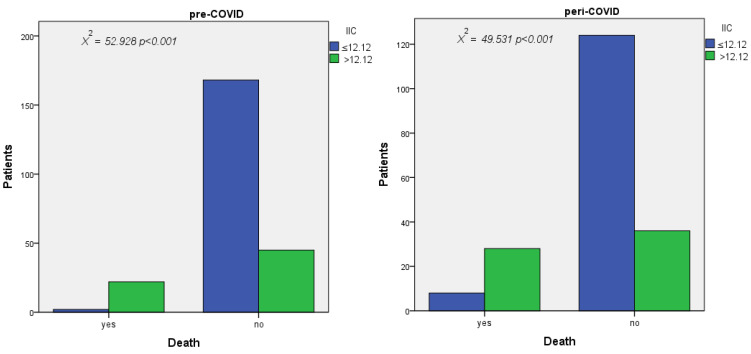
Correlation between mortality and IIC in the pre-COVID andperi-COVID group. *X^2^*—chi-square value.

As IIC predicted death very well in both groups, with close cutoff values, we selected its highest cutoff value as a reference. We used this value to determine the link between death and the IIC level (Figure 6) by performing the Pearson chi-square test, in which we tested the occurrence of death in the groups with a low IIC and high IIC for each period. We obtained a statistically significant result (*p* < 0.001) with an odds ratio (risk) of 4.08 in the pre-COVID group and 3.33 in the peri-COVID group. The results of the Pearson chi-square test were significant (*p* < 0.001), showing that patients with an IIC value >12.12 had a 4.08-fold risk of dying in the pre-COVID group and a 3.33-fold risk in the peri-COVID group, respectively, compared to patients with an IIC value ≤ 12.12 (Table 13).

### Limitations of the study

First of all, the study design is a retrospective one. Another limitation would be the impossibility of excluding the impact of some treatments before hospitalization on the inflammatory index values. Another limitation of the study would be the fact that we did not compare inflammatory markers with other biochemical markers such as procalcitonin or IL-6. Moreover, another limitation would be the fact that we did not describe the changes in inflammatory markers that could appear later during hospitalization following the treatment performed, a fact that could better estimate the prognosis of the patient with PA.

## 5. Conclusions

In conclusion, the MCVL had a significant predictive value in complications with surgical risk (abscess, necrosis, and pseudocyst), and IIC had the highest predictive value regarding mortality, both in the pre-COVID and in the peri-COVID period, being more predictive than the already known inflammatory markers that registered a specificity below 50% in the peri-COVID group. The results show that the PA patients who had an IIC > 12.12 presented a risk of dying that was 4.08 times higher in the pre-COVID group and 3.33 times in the peri-COVID group, respectively, compared to patients with an IIC value of ≤ 12.12. MCV and IIC are cheap, easy to evaluate, available in every healthcare center, and following the results obtained, they can be applied on a large scale in populations with altered immune and inflammatory status. Future clinical research should take into account the increased values of these two markers in order to achieve a better stratification of patients with acute pancreatitis and the application of improved treatment to reduce complications and mortality.

## Figures and Tables

**Table 1:** Research questions.

Research Questions
Did the number of patients diagnosed with AP decrease during the COVID-19 pandemic, and what would the explanation for this be? Did the mean values of the already known inflammatory markers NLR, PLR, MLR, dNLR, AISI, SIRI, and SII and the newly introduced MCVL and IIC change during the COVID-19 period? Were there any differences in terms of the number of complications with surgical risk and their degree of operability during the COVID-19 period? Among the already known inflammatory markers NLR, PLR, MLR, dNLR, AISI, SIRI, and SII and the newly introduced MCVL and IIC, which of them can effectively predict the complications with the surgical risk (abscess, necrosis, and pseudocyst) and mortality in the pre-COVID and peri-COVID periods?

**Table 2:** Demographic characteristics of pre-COVID peri-COVID patients.

Variable	Category	Total n = 433	Pre-COVID n = 237	Peri-COVID n = 196	*p*
Gender	Men	225 (51.9%)	134 (56.5%)	91 (46.4%)	0.036 *
	Women	208 (48.03)	103 (43.5%)	105 (53.6%)	
Age	Men				
	mean ± SD Women	59.44 ± 15.21	57.57 ± 13.43	62.19 ± 17.21	0.596
	mean ± SD	62.2 ± 18.07	58.78 ± 19.89	65.55 ± 15.45	0.151
	Total				
	mean ± SD	60.76 ± 16.68	58.09 ± 19.52	63.99 ± 16.34	<0.001 *
Etiology	Biliary	357 (82.4%)	195 (82.2%)	162 (82.6%)	0.830
	Alcohol	53 (12.2%)	31 (13%)	22 (11.2%)	
	Unknown	23 (5.3%)	11 (4.6%)	12 (6.1%)	
Form	Mild	120 (27.7%)	81 (34.1%)	39 (19.8%)	0.072
	Moderate	184 (42.4%)	86 (36.2%)	98 (50.0%)	
	Severe	129 (29.7%)	70 (29.5%)	59 (30.1%)	
Hours_onset		35.28 ± 26.4	32.99 ± 26.27	38.04 ± 26.34	0.047 *
Hosp_days		12.81 ± 13.31	14 ± 16.55	11.37 ± 7.58	0.030 *
Complications	Abscess	24 (5.5%)	9 (3.8%)	15(7.7%)	0.006 *
	Necrosis	29 (6.7%)	9 (3.8%)	20 (10.2%)	
	Pseudocyst	15 (3.5%)	10 (4.2%)	5 (2.6%)	
	Without	364 (84.1%)	208 (87.8%)	156 (79.6%)	
Treatment	Surgical	25 (5.8%)	16 (6.8%)	9 (4.6%)	0.339
	Medical	408 (94.2%)	221 (93.2%)	187 (95.4%)	
Mortality	Discharged	60 (13.9%)	24 (10.1%)	36 (18.4%)	0.016 *
	Alive	373 (86.1%)	213 (89.9%)	160 (81.6%)	

* *p* < 0.05—statistically significant.

**Table 3:** Demographic characteristics of patients with AP and COVID-19 in the peri-COVID group.

Variable	Category	Peri-COVID n = 196	COVID-19 n = 28	Non-COVID-19 n = 168	*p*
Gender	Men	91 (46.4%)	19 (67.9%)	72 (32.8%)	0.017 *
	Women	105 (53.5%)	9 (32.1%)	96 (67.2%)	
Age	Men				
	mean ± SD Women	62.19 ± 17.21	47 ± 15.61	66.19 ± 15.35	<0.001 *
	mean ± SD	65.55 ± 15.45	48.44 ± 20.71	67.16 ± 13.96	<0.001 *
	Total				
	mean ± SD	63.99 ± 16.34	47.46 ± 17.03	66.74 ± 14.54	<0.001 *
Etiology	Biliary	162 (82.6%)	13 (46.4%)	149 (88.7%)	<0.001 *
	Alcohol	22 (11.22%)	8 (28.6%)	14 (8.3%)	
	Unknown	12 (6.12%)	7 (25%)	5 (3%)	
Form	Mild	39 (30.1%)	3 (10.7%)	117 (28.9%)	0.040 *
	Moderate	98 (50%)	13 (46.4%)	171 (42.2%)	
	Severe	59 (30.1%)	12 (42.9%)	117 (28.9%)	
Hours_onset		38.04 ± 26.34	41 ± 32.27	37.55 ± 25.30	0.594
Hosp_days		11.37 ± 7.58	12.36 ± 8.89	11.21 ± 7.36	0.459
Complications	Abscess	15 (7.7%)	3 (10.7%)	12 (7.1%)	0.142
	Necrosis	20 (10.2%)	5 (17.9%)	15 (8.9%)	
	Pseudocyst	6 (3.1%)	2 (7.1%)	4 (2.4%)	
	Without	155 (79.1%)	18 (64.3%)	137 (81.5%)	
Treatment	Surgical	10 (4.6%)	1 (3.6%)	9 (5.4%)	0.693
	Medical	186 (95.4%)	27 (96.4%)	159 (94.6%)	
Mortality	Discharged	36 (18.4%)	12 (42.9%)	24 (14.3%)	0.007 *
	Alive	160 (81.6%)	16 (57.1%)	144 (85.7%)	

* *p* < 0.05—statistically significant. The *p*-value results from the comparison of the group of COVID-19 patients with the group of non-COVID-19 patients.

**Table 4:** Comparison of laboratory parameters and inflammatory markers between pre-COVID and peri-COVID groups in PA patients.

Laboratory Parameters	Pre-COVID n = 237	Peri-COVID n = 196	*p*	COVID-19 n = 28	Non-COVID-19 n = 168	*p*
WBC (×10^3^/μL)	13.25 ± 5.84	13.64 ± 6.71	0.047 *	16.07 ± 7.94	13.24 ± 6.42	0.082
NEU (×10^3^/μL)	10.81 ± 5.86	11.17 ± 6.45	0.513	12.07 ± 7.48	11.02 ± 6.27	0.427
LYM (×10^3^/μL)	1.65 ± 0.94	1.53 ± 1.26	0.545	1.99 ± 1.18	1.45 ± 1.26	0.037 *
MON (×10^3^/μL)	0.86 ± 0.48	0.97 ± 1.21	0.242	1.84 ± 2.83	0.82 ± 0.51	0.069
PLT (×10^3^/μL)	217.50 ± 115.79	212.97 ± 113.79	0.683	237.43 ± 143.72	208.89 ± 107.99	0.220
MCV (fL)	89.67 ± 8.68	93.57 ± 7.56	<0.001 *	96.02 ± 7.85	93.17 ± 7.46	0.065
RDW	13.24 ± 1.88	13.51 ± 1.48	0.101	14.20 ± 1.36	13.39 ± 1.47	0.007 *
NLR	8.46 ± 6.01	10.24 ± 8.61	0.015 *	7.85 ± 6.17	10.64 ± 8.91	0.045 *
PLR	160.35 ± 112.62	171.72 ± 105.24	0.282	114.23 ± 112.56	176.30 ± 103.62	0.136
MLR	0.61 ± 0.38	0.78 ± 0.77	0.005 *	1.04 ± 1.41	0.74 ± 0.60	0.278
dNLR	4.66 ± 2.93	6.08 ± 6.68	0.006 *	4.40 ± 3.34	6.36 ± 7.05	0.152
AISI	1899.28 ± 2994.76	2075.22 ± 280372	0.531	2352.12 ± 2240.83	2029.07 ± 2889.99	0.574
SIRI	7.76 ± 8.13	9.28 ± 10.98	0.109	10.63 ± 11.35	9.06 ± 10.93	0.438
SII	1870.92 ± 2000.38	2072.69 ± 2193.28	0.318	1689.64 ± 1460.38	2136.53 ± 2289.87	0.319
MCVL	70.26 ± 37.17	90.65 ± 67.15	<0.001 *	65.04 ± 34.33	94.91 ± 70.33	0.029 *
IIC	10.73 ± 8.71	13.03 ± 10.86	0.006 *	11.04 ± 8.99	13.36 ± 11.31	<0.001 *

* *p* < 0.05—statistically significant; WBC—leukocytes, NEU—neutrophils, LYM—lymphocytes, MON—monocytes, PLT—platelets, MCV—mean corpuscular volume, RDW—erythrocyte distribution width, NLR—neutrophil–lymphocyte ratio, PLR—platelet–lymphocyte ratio, MLR—monocyte–lymphocyte ratio, dNLR—derived neutrophil–lymphocyte ratio, AISI—the aggregate index of systemic inflammation, SIRI—systemic inflammatory response index, SII—systemic immune-inflammation index, MCVL—average corpuscular volume to lymphocytes ratio, IIC—cumulative inflammatory index.

**Table 5:** Comparison of patients parameters according to complications and mortality in the pre-COVID group.

Laboratory Parameters	With Complications	Without Complications	*p*	Deceased n = 24	Alive n = 213	*p*
Gender (M/F)	20/8 (71.4%/28.6%)	106/103 (50.7%/49.3%)	0.039 *†	10/14 41.7%/58.3%	124/89 58.3%/41.8%	0.121 †
Age	52.4 ± 2.54	58.9 ± 1.15	0.072	63.25 ± 1.67	57.51 ± 1.17	0.007 *
Hours_onset	42.86 ± 5.43	31.67 ± 1.77	0.212	44.17 ± 6.56	31.73 ± 1.73	0.078
Area (U/R)	13/15 (46.4%/53.6%)	121/88 (57.9%/42.1%)	0.250 †	10/14 41.7%/58.3%	124/89 58.3%/41.8%	0.121 †
Proteins	6.24 ± 0.10	6.37 ± 0.05	<0.001 *	5.83 ± 0.14	6.42 ± 0.49	<0.001 *
Amylase	373.76 ± 29.99	462.19 ± 42.17	0.694	492.54 ± 89.89	448.06 ± 40.90	0.721
AST	67.36 ± 45.85	142.83 ± 237.83	0.001 *	100.13 ± 135.31	123.3 ± 208.16	0.466
ALT	57.90 ± 58.53	155.91 ± 222.78	0.001 *	92.33 ± 77.44	152.00 ± 222.76	0.008 *
BT	2.06 ± 4.26	1.76 ± 2.23	0.573	4.19 ± 5.62	1.51 ± 1.66	0.029 *
Urea	47.95 ± 4.1	53.49 ± 3.04	0.48	92.12 ± 10.91	48.23 ± 2.58	0.001 *
Creatinine	1.19 ± 0.16	1.35 ± 0.09	<0.001 *	2.54 ± 0.36	1.19 ± 0.08	0.001 *
Glucose	159.24 ± 14.30	109.55 ± 4.02	0.330	100.46 ± 6.87	117.4 ± 4.49	0.186
INR	2.22 ± 0.56	1.23 ± 0.02	0.087	2.7 ± 0.64	1.19 ± 0.08	0.028 *
Hb (g/dl)	13.27 ± 0.55	13.01 ± 0.17	0.213	11.41 ± 0.36	13.23 ± 0.18	0.001 *
Ht (%)	39.28 ± 1.44	38.71 ± 0.50	<0.001 *	34.47 ± 1.04	39.27 ± 0.50	0.002 *
WBC (×10^3^/μL)	13.85 ± 6.92	13.17 ± 5.70	0.564	14.52 ± 5.93	13.1 ± 5.83	0.259
NEU (×10^3^/μL)	11.58 ± 6.52	10.71 ± 5.78	0.459	14.38 ± 7.48	10.41 ± 5.53	0.002 *
LYM (×10^3^/μL)	1.16 ± 0.39	1.72 ± 0.79	<0.001 *	0.91 ± 0.41	1.74 ± 0.94	<0.001 *
MON (×10^3^/μL)	0.99 ± 0.45	0.84 ± 0.48	0.122	0.90 ± 0.51	0.85 ± 0.48	0.672
PLT (×10^3^/μL)	245.32 ± 194.70	213.77 ± 100.85	0.407	207.89 ± 26.22	218.58 ± 114.55	0.669
MCV (fL)	89.85 ± 8.95	89.64 ± 8.67	0.908	87.83 ± 8.26	89.87 ± 8.72	0.277
RDW	13.52 ± 1.54	13.20 ± 1.92	0.392	14.5 ± 1.30	13.09 ± 1.88	<0.001 *
NLR	10.41 ± 4.85	8.20 ± 6.10	0.067	17.609 ± 7.15	7.43 ± 4.9	<0.001 *
PLR	206.73 ± 108.62	154.13 ± 111.95	0.020 *	227.36 ± 119	152.8 ± 109.63	0.002 *
MLR	0.92 ± 0.48	5.03 ± 2.04	0.001 *	0.96 ± 0.45	0.57 ± 0.35	<0.001 *
dNLR	5.03 ± 2.04	4.61 ± 3.03	0.484	8.06 ± 4.82	4.28 ± 2.36	0.001 *
AISI	3676.43 ± 4063.20	1661.20 ± 2747.85	0.016 *	3891.86 ± 4342.95	1674.77 ± 2727.18	0.022 *
SIRI	11.73 ± 8.02	7.23 ± 8.01	0.006 *	14.34 ± 9.69	7.02 ± 7.61	0.001 *
SII	2963.02 ± 2910.07	1724.61 ± 1805.22	0.036 *	3542.55 ± 2775.46	1682.57 ± 1807.43	0.004 *
MCVL	84.69 ± 25.93	13.01 ± 6.75	0.028 *	117.88 ± 52.93	64.89 ± 30.80	<0.001 *
IIC	13.01 ± 6.75	10.01 ± 8.89	0.088	22.62 ± 10.55	8.99 ± 7.31	<0.001 *

* *p* < 0.05—statistically significant † Chi-square test; AST—aspartate aminotransferase, ALT—alanine aminotransferase, BT—total bilirubin, WBC—leukocytes, NEU—neutrophils, LYM—lymphocytes, MON—monocytes, PLT—platelets, MCV—mean corpuscular volume, RDW—erythrocyte distribution width, NLR—neutrophil–lymphocyte ratio, PLR—platelet–lymphocyte ratio, MLR—monocyte–lymphocytes ration, dNLR—derived neutrophil–lymphocyte ratio, AISI—aggregate index of systemic inflammation, SIRI—systemic inflammatory response index, SII—systemic immune-inflammation index, MCVL—average corpuscular volume–lymphocytes ratio, IIC—cumulative inflammatory index.

**Table 6:** Comparison of patients parameters according to complications and mortality in the peri-COVID group.

Laboratory Parameters	With Complications	Without Complications	*p*	Deceased n = 36	Alive n = 213	*p*
Gender (M/F)	21/19 (52.5%/47.5%)	106/103 (52.5%/47.5%)	0.388 †	21/15 58.3%/41.7%	70/90 43.8%/56.3%	0.113 †
Age	61.73 ± 2.74	64.57 ± 1.28	0.561	63.08 ± 2.73	64.19 ± 1.29	0.714
Hours_onset	46.75 ± 4.96	35.81 ± 1.96	0.091	44.08 ± 4.31	36.68 ± 2.08	0.128
Area (U/R)	12/28 30%/70%	85/71 54.5%/45.5%	0.006 *†	15/21 41.7%/58.3%	82/78 51.2%/48.8%	0.299 †
Proteins	5.89 ± 0.14	6.36 ± 0.07	<0.001 *	5.81 ± 0.15	6.37 ± 0.06	0.001 *
Amylase	598.03 ± 100.13	597.99 ± 58.47	0.513	758.03 ± 105.27	561.53 ± 57.18	0.132
AST	142.83 ± 237.83	57.90 ± 58.53	0.910	252.17 ± 343.29	97.56 ± 106.92	0.023 *
ALT	138.21 ± 197.66	163.05 ± 188.02	<0.001 *	113.73 ± 149.93	151.50 ± 185.64	0.022 *
BT	2.86 ± 4.76	2.37 ± 3.71	0.494	5.61 ± 7.13	1.75 ± 2.20	0.003 *
Urea	76.95 ± 8.85	50.94 ± 3.73	0.600	95.17 ± 13.47	47.45 ± 2.66	0.001 *
Creatinine	2.67 ± 0.41	1.2 ± 0.10	0.664	3.35 ± 0.48	1.08 ± 0.07	<0.001 *
Glucose	145.93 ± 16.02	126.63 ± 5.34	0.010 *	185.39 ± 18.24	47.45 ± 2.66	0.001 *
INR	1.26 ± 0.04	1.21 ± 0.03	0.924	1.39 ± 0.06	1.18 ± 0.02	0.001 *
COVID-19	9/32.1%	31/18.5%	0.097†	12/42.9%	24/14.3%	<0.001 *†
Hb (g/dl)	12.15 ± 0.45	13.10 ± 0.16	0.044	11.77 ± 0.55	13.16 ± 0.15	0.019 *
Ht (%)	36 ± 1.23	38.5 ± 0.61	0.364	34.17 ± 1.40	38.85 ± 0.57	0.003 *
WBC (×10^3^/μL)	13.18 ± 6.75	10.01 ± 8.89	0.629	15.82 ± 9.63	13.15 ± 5.78	0.118
NEU (×10^3^/μL)	11.09 ± 6.01	11.19 ± 6.57	0.927	13.86 ± 8.94	10.57 ± 5.60	0.040 *
LYM (×10^3^/μL)	1.18 ± 0.84	1.62 ± 1.34	0.050	0.99 ± 0.50	1.65 ± 1.35	<0.001 *
MON (×10^3^/μL)	0.84 ± 0.37	1 ± 1.34	0.458	0.85 ± 0.44	0.99 ± 1.32	0.519
PLT (×10^3^/μL)	191.69 ± 124.72	218.42 ± 110.58	0.186	168.59 ± 125.62	22.95 ± 108.90	0.009 *
MCV (fL)	99.63 ± 5.12	92.02 ± 7.31	<0.001 *	103.46 ± 4.70	91.35 ± 6.17	<0.001 *
RDW	13.83 ± 1.79	13.43 ± 1.39	0.191	14.57 ± 1.9	13.27 ± 1.26	<0.001 *
NLR	11.49 ± 7.15	9.92 ± 8.95	0.305	14.86 ± 7.72	9.20 ± 8.49	<0.001 *
PLR	171.01 ± 74.68	171.90 ± 111.94	0.962	175.99 ± 105.89	170.76 ± 105.41	0.788
MLR	0.94 ± 0.66	0.74 ± 0.79	0.138	1.07 ± 0.72	0.71 ± 0.77	0.011 *
dNLR	8.20 ± 12.50	5.53 ± 3.92	0.191	7.18 ± 4.42	5.83 ± 7.08	0.275
AISI	1738.37 ± 1569.47	2161.59 ± 3078.57	0.226	2604.11 ± 2810.62	1956.22 ± 2797.18	0.211
SIRI	8.84 ± 4.75	9.39 ± 12.07	0.654	12.98 ± 9.39	8.45 ± 11.16	0.025 *
SII	1865.62 ± 1255.04	2125.78 ± 2375.22	0.505	2477.51 ± 2015.04	1981.60 ± 2227.2	0.221
MCVL	128.53 ± 99.54	80.93 ± 52.10	0.005 *	146.64 ± 104.99	78.05 ± 47.27	<0.001 *
IIC	17.20 ± 10.28	12.20 ± 10.73	0.009 *	12.12 ± 9.24	10.99 ± 9.86	<0.001 *

* *p* < 0.05—statistically significant. † Chi-square test. AST—aspartate aminotransferase, ALT—alanine aminotransferase, BT—total bilirubin, WBC—leukocytes, NEU—neutrophils, LYM—lymphocytes, MON—monocytes, PLT—platelets, MCV—mean corpuscular volume, RDW—erythrocyte distribution width, NLR—neutrophil–lymphocyte ratio, PLR—platelet–lymphocyte ratio, MLR—monocyte–lymphocyte ratio, dNLR—derived neutrophil–lymphocyte ratio, AISI—aggregate index of systemic inflammation, SIRI—systemic inflammatory response index, SII—systemic immune-inflammation index, MCVL—average corpuscular volume–lymphocytes ratio, IIC—cumulative inflammatory index.

**Table 7:** Cutoff values of laboratory parameters for determining the risk of complications with surgical risk in the pre-COVID (1) and peri-COVID (2) groups.

Variable		AUC (95%)	Lowest Value	Highest Value	Cutoff	*p*	Sensitivity	Specificity
NLR	1 2	0.651 0.616	0.545 0.535	0.756 0.697	5.58 6.41	0.010 * 0.023 *	78.6% 80%	42.2% 44.9%
PLR	1 2	0.657 0.678	0.545 0.487	0.768 0.668	97.3 140.27	0.007 * 0.130	89.3% 72.5%	34.5% 50.6%
MLR	1 2	0.719 0.639	0.602 0.550	0.837 0.729	0.66 0.53	<0.001 * 0.007 *	78.6% 75%	69.9% 49.4%
dNLR	1 2	0.600 0.534	0.494 0.448	0.706 0.619	2.82 3.12	0.085 0.518	78.6% 80%	29.1% 32.1%
AISI	1 2	0.635 0.532	0.497 0.439	0.774 0.625	228.79 358.98	0.020 * 0.533	78.6% 92.5%	9.2% 21.2%
SIRI	1 2	0.672 0.619	0.548 0.534	0.797 0.703	3.56 2.49	0.003 * 0.021 *	78.6% 92.5%	44.2% 25%
SII	1 2	0.620 0.537	0.490 0.445	0.749 0.630	507.7 756.8	0.040 * 0.468	89.3% 92.5%	17% 26.9%
MCVL	1 2	0.697 0.681	0.609 0.591	0.785 0.771	64.89 78	0.001 * <0.001 *	78.6% 80%	56.8% 60.3%
IIC	1 2	0.663 0.686	0.554 0.605	0.772 0.767	8.41 10.51	0.005* <0.001 *	78.6% 72.5%	55.8% 52.6%

* *p* < 0.05—statistically significant; NLR—neutrophil–lymphocyte ratio, PLR—platelet–lymphocyte ratio, MLR—monocyte–lymphocyte ratio, dNLR—derived neutrophil–lymphocyte ratio, AISI—aggregate index of systemic inflammation, SIRI—systemic inflammatory response index, SII—systemic immune-inflammation index, MCVL—average corpuscular volume–lymphocyte ratio, IIC—cumulative inflammatory index.

**Table 8:** Cutoff values of laboratory parameters for determining the risk of death in the pre-COVID (1) and peri-COVID (2) groups.

Variable		AUC(95%)	Lowest Value	Highest Value	Cutoff	*p*	Sensitivity	Specificity
NLR	1 2	0.833 0.743	0.811 0.662	0.954 0.824	11.01 5.93	<0.001 * <0.001 *	91.7% 91.7%	80.5% 42.5%
PLR	1 2	0.692 0.557	0.569 0.451	0.816 0.663	102.88 100.38	0.002 * 0.287	87.5% 86.1%	38.1% 25.6%
MLR	1 2	0.737 0.680	0.618 0.575	0.855 0.785	0.38 0.36	<0.001 * 0.001 *	87.5% 86.1%	29.5% 28.1%
dNLR	1 2	0.782 0.673	0.681 0.587	0.884 0.759	3 3.53	<0.001 * 0.001 *	87.5% 91.7%	35.2% 43.1%
AISI	1 2	0.632 0.536	0.506 0.417	0.758 0.656	360.26 433.57	0.034 * 0.495	87.5% 86.1%	26.7% 23.1%
SIRI	1 2	0.754 0.688	0.657 0.589	0.851 0.787	4.26 2.85	<0.001 * <0.001 *	87.5% 86.1%	49% 26.9%
SII	1 2	0.747 0.561	0.629 0.44	0.864 0.681	906.21 756.8	<0.001 * 0.256	87.5% 86.1%	41% 25%
MCVL	1 2	0.817 0.762	0.739 0.684	0.896 0.84	72.14 74.9	<0.001 * <0.001 *	91.7% 94.4%	67.6% 57.5%
IIC	1 2	0.887 0.870	0.819 0.815	0.956 0.926	13.29 12.12	<0.001 * <0.001 *	91.7% 91.7%	78.6% 72.5%

* *p* < 0.05—statistically significant. NLR—neutrophil–lymphocyte ratio, PLR—platelet–lymphocyte ratio, MLR—monocyte–lymphocyte ratio, dNLR—derived neutrophil–lymphocyte ratio, AISI—aggregate index of systemic inflammation, SIRI—systemic inflammatory response index, SII—systemic immune-inflammation index, MCVL—average corpuscular volume–lymphocyte ratio, IIC—cumulative inflammatory index.

**Table 9:** Univariate and multivariate analyses of variables associated with complications in pre-COVID group.

Variable	Univariate Analysis	*p* Value	Multivariate Analysis	*p* Value
	OR		OR	
Proteins	1.29 (0.75–2.21)	0.347		
Creatinine	1.12 (0.77–1.62)	0.547		
AST	1.004 (0.998–1.010)	0.153		
ALT	1.005(1.000–1.009)	0.062		
Ht	0.99 (0.94–1.04)	0.703		
LYM	3.33 (1.15–7.32)	0.003 *	0.05 (0.004–0.785)	0.032 *
PLR				
≤97.3 (Ref)				
>97.3	4.56 (1.33–15.63)	0.016 *	2.04 (0.53–7.38)	0.296
MLR				
≤0.66 (Ref)				
>0.66	8.69 (3.36–22.48)	<0.001 *	0.43 (0.19–0.99)	0.048 *
AISI				
≤228.79 (Ref)				
>228.79	0.83 (0.23–3.01)	0.781		
SIRI				
≤3.56 (Ref) >3.56	2.99 (1.16–7.69)	0.023 *	2.38 (0.90–6.22)	0.077
SII				
≤507.7 (Ref)				
>507.7	1.67 (0.48–5.85)	0.418		
MCVL				
≤64.89 (Ref)				
>64.89	4.94 (1.92–12.69)	<0.001 *	3.52 (1.52–8.13)	0.003 *
IIC				
≤8.41 (Ref)				
>8.41	5.56 (2.87–10.95)	<0.001 *	2.80 (1.00–7.84)	0.049 *

* *p* < 0.05—statistically significant.

**Table 10:** Univariate and multivariate analyses of variables associated with complications in peri-COVID group.

Variable	Univariate Analysis	*p* Value	Multivariate Analysis	*p* Value
	OR		OR	
Area	0.35 (0.17–0.75)	0.007 *	0.36 (0.15–0.89)	0.027 *
Proteins	1.88 (1.22–2.92)	0.004 *	1.25 (0.76–2.06)	0.360
Glucose	0.99 (0.992–1.001)	0.150		
ALT	1.011(1.004–1.018)	0.001 *	1.008 (1.001–1016)	0.026 *
MCV	0.84 (0.79–0.90)	<0.001 *	0.86 (0.80–0.92)	<0.001 *
MCVL				
≤78 (Ref)				
>78	3.51 (1.52–8.11)	<0.001 *	4.22 (1.46–12.14)	0.008 *
IIC				
≤10.51 (Ref)				
>10.51	3.64 (2.07–6.38)	<0.001 *	1.60 (0.66–3.88)	0.295

* *p* < 0.05—statistically significant.

**Table 11:** Univariate and multivariate analyses of variables associated with mortality in pre-COVID group.

Variable	Univariate Analysis	*p* Value	Multivariate Analysis	*p* Value
	OR		OR	
Age	0.97 (0.95–1)	0.109		
Proteins	3.11 (1.66–5.82)	0.001 *	0.97 (0.20–4.53)	0.970
Urea	0.98 (0.97–0.99)	<0.001 *	0.96 (0.93–0.99)	0.013 *
Creatinine	0.63 (0.49–0.811)	<0.001 *	1.24 (0.64–2.39)	0.517
ALT	1.002 (0.999–1.006)	0.212		
BT	0.75 (0.65–0.87)	<0.001 *	0.75 (0.59–0.92)	0.009 *
INR	0.13 (0.04–0.41)	0.001 *	0.48 (0.21–1.09)	0.083
Hb	1.46 (1.18–1.82)	0.001 *	2.56 (0.50–13.10)	0.258
Ht	1.12 (1.04–1.21)	0.001 *	0.75 (0.59–0.92)	0.980
NEU	0.90 (0.85–0.96)	0.003 *	0.85 (0.74–0.98)	0.025 *
LYM	19.86 (5.15–76.49)	<0.001 *	7.46 (1.51–36.77)	0.013 *
RDW	0.73 (0.59–0.89)	0.003 *	0.70 (0.47–1.05)	0.086
NLR				
≤11.01 (Ref)				
>11.01	46.14 (10.43–204.15)	<0.001 *	20.10 (3.12–129.42)	0.002 *
PLR				
≤102.88 (Ref)				
>102.88	4.46 (1.29–15.45)	0.018 *	0.50 (0.10–2.45)	0.399
MLR				
≤0.38 (Ref)				
>0.38	2.87 (0.82–9.98)	0.097		
dNLR				
≤3 (Ref)				
>3	4.21 (1.21–14.56)	0.023 *	0.63 (0.12–3.21)	0.582
AISI				
≤360.26 (Ref)				
>360.26	2.55 (0.73–8.90)	0.140		
SIRI				
≤4.26 (Ref)				
>4.26	6.93 (2.00–23.94)	0.002 *	0.83 (0.51–1.35)	0.458
SII				
≤906.21 (Ref)				
>906.21	5.02 (1.45–17.36)	0.011 *	7.64 (0.62–94.05)	0.112
MCVL				
≤72.14 (Ref)				
>72.14	23.45 (5.36–102.62)	<0.001 *	5.28 (0.90–30.73)	0.064
IIC				
≤13.29 (Ref)				
>13.29	41.06 (9.30–181.2)	<0.001 *	18.71 (2.60–134.52)	0.004 *

* *p* < 0.05—statistically significant.

**Table 12:** Univariate and multivariate analyses of variables associated with mortality in peri-COVID group.

Variable	Univariate Analysis	*p* Value	Multivariate Analysis	*p* Value
	OR		OR	
Age	1 (0.98–1.02)	0.712		
Proteins	2.21 (1.37–3.55)	0.001 *	9.16 (1.72–48.75)	0.009 *
Urea	0.98 (0.97–0.99)	<0.001 *	1.03 (1.00–1.07)	0.015 *
Creatinine	0 (0.39–0.67)	<0.001 *	0.07 (0.01–0.30)	<0.001 *
AST	0.998 (0.996–0.999)	0.002 *	1.001 (0.997–1.005)	0.586
ALT	1.003 (0.999–1.006)	0.103		
BT	0.81 (0.73–0.90)	<0.001 *	0.71 (0.57–0.89)	0.003 *
INR	0.23 (0.09–0.59)	0.002 *	12.09 (11.97–12.22)	0.042
COVID-19	4.5 (1.89–10.68)	0.001 *	53.75 (4.96–581.85)	0.001 *
Hb	1.32 (1.11–1.56)	0.001 *	0.55 (0.21–1.46)	0.232
Ht	1.07 (1.02–1.12)	0.002 *	1.2 (0.91–1.56)	0.181
NEU	0.93 (0.88–0.98)	0.007 *	0.71 (0.57–0.88)	0.002 *
LYM	3.81 (1.85–7.85)	<0.001 *	1.86 (0.62–5.53)	0.261
RDW	0.58 (0.46–0.75)	<0.001 *	0.85 (0.45–1.61)	0.634
NLR				
≤5.93 (Ref)				
>5.93	8.13 (2.39–27.61)	0.001 *	10.24 (1.29–81.17)	0.028 *
MLR				
≤0.36 (Ref)				
>0.36	1.95 (0.76–5.01)	0.163		
SIRI				
≤2.85 (Ref)				
>2.85	2.27 (0.83–6.23)	0.109		
MCVL				
≤74.9 (Ref)				
>74.9	23 (5.34–99.04)	<0.001 *	8.92 (5.21–141.58)	0.041 *
IIC				
≤12.12 (Ref)				
>12.12	29 (8.46–99.39)	<0.001 *	27.94 (3.57–218.58)	0.002

* *p* < 0.05—statistically significant.

**Table 13:** Chi-square analysis of the risk of complications and mortality of IIC and MCVL in the pre-COVID and peri-COVID period and the adjusted cutoff value of the IIC in the pre-COVID group.

Pre-COVID						Peri-COVID				
	Cutoff	OR	Chi-Square	df	*P*	Cutoff	OR	Chi-Square	df	*p*
**Death**										
IIC		41.067	52.928	1	<0.001*		29.000	50.729	1	<0.001*
Cutoff **ROC**	>13.29	4.339				**>12.12**	3.333			
	≤13.29	0.106				**≤12.12**	0.115			
IIC Adjusted	**>12.12**	37.813 4.086	49.531	1	<0.001*				1	<0.001*
Cutoff	**≤12.12**	0.108								
**Complications**										
MCVL		4.944	12.843	1	<0.001*		6.065	20.672	1	<0.001*
Cutoff ROC	>64.89	1.845				>78	2.013			
	≤64.89	0.373				≤78	0.332			

* *p* < 0.05—statistically significant. “OR–ROC”—cutoff adjusted from ROC curve. “ICC adjusted”—ICC adjusted from the IIC value of higher OR obtained after multivariate analysis. The bold cutoff value is the one that maintained the increased OR value after the multivariate analysis.

## Data Availability

Not applicable.
